# On the Treatment of Cholera

**Published:** 1849-01

**Authors:** 


### Part 3.—Selections.

### ARTICLE I.

*On the Treatment of Cholera.*

(Monthly Journal, March 1848.) In a review of Parkes, Milroy, Giacomini and Cowdell on Cholera, we find the following remarks:

It is unnecessary, perhaps, for us to remark, that every possible kind of remedy, and plan of treatment, even the most opposite in their nature, have been proposed and employed in Asiatic cholera. This at once betrays the absence of rational indications based upon a knowledge of the pathology of the disease. It is not, then, our intention to draw up a catalogue of what the numerous individual experiences and ideas of practitioners have led them to recommend, but rather to place before our readers the views of our authors on the subject, and ascertain, if possible, how far they may reasonably be considered consistent with the known phenomena the dsease presents.

According to Dr. Parkes, cholera runs a certain course. When the algide symptoms have once shown themselves, a case cannot be cut short. Even in the mildest forms warmth does not return for a long time; but, when the disease has reached its acme, the patient is invariably seen to remain in a peculiar state, during which time nature seems gradually to be repairing the injury which has been done. If respiration could be maintained—not the mere mechanical act of breathing in and out, but the chemical process in sufficient integrity to allow the blood to circulate through the capillaries of the lungs—nature would gradually bring about the cure. This is the great problem which medicine has to accomplish, and which, next to the discovery of some actual antidote to the poison itself, appears to be the most ready method of accomplishing the cure of cholera.—(*Parkes, p.* 204.)

According to Giacomini, the rational treatment consists in overcoming the phlebitis (venus congestion,) causing suspension of the circulation. For this purpose various hyposthenics are indicated; but he says they are often useless, because there is a complete absence of assimilation in cholera patients.

The instantaneous dryness of the cellular tissue by the operation of the morbid matter absorbed, and the filled state of the veins are such, that at a certain epoch of the disease, there are no means of causing any remedy whatever to pass by assimilation into the blood. The skin is as if dead, and does not absorb, and what is introduced into the stomach only washes or encumbers the absorbing passages. Consequently the best indicated remedies are not digested or absorbed, the digestive organs and skin not lending themselves to this office, and the most powerful resources of art are rendered of no effect, in fault of a proper channel whereby they may be introduced into the blood.—*Annales, p.* 333.

Dr. Parkes makes exactly the same statement, saying—

The great difficulty in the treatment of cholera, and the cause of the contradictory and opposing statements which have been made respecting the value of particular medicines, is to be found in the peculiar action of the choleraic poison. This action by arresting the circulation, and thereby rendering absorption dfficult, opposing itself to the common method of administering remedies. After a certain period of the disease, medicines remain in the stomach, and do not pass into the circulation, or do so with great difficulty and slowness. At least this is to be inferred, both from the circumstance that in the advanced stage, calomel, acetate of lead, kreosote, opium, turpentine, &c., have been found in the stomach hours after they have been taken, and that fluids taken to appease thirst, remain in and distend the stomach, if they are not vomited, and also from the evident languor and delay of the circulation,—states which are considered unfavorable for absorption. —p. 200.

As medicines, therefore, cannot with any good effect be given internally, other means must be adopted for overcoming the congestion. Of these the most powerful seems to be bleeding, cold to the surface, and injections into the veins. Let us examine what our authors tell us regarding each of these means of cure.

*Bleeding.*—Dr. Parkes states that the benefit resulting from bleeding was generally more marked according as the disease was in its earliest stage, and according as it tended towards the several varieties of pseudo-cholera.

In these latter cases the employment of blood-letting was sometimes followed by very striking results, particularly in those cases attended by a full pulse, and severe general spasms.

For example, I saw a stout European soldier one hour after admission into the hospital: he was violently purged and vomited, and laboring under the most severe and frightful spasms. They were general and quite tetanic in character; the pulse was hard and sharp; the skin was warm. He had been treated with calomel and opium without benefit. I immediately opened a vein, and took away forty ounces of blood before the spasms ceased. I then gave him Tinct Opii, ℨj. and repeated it in an hour. The pulse immediately after the bleeding became fuller and less resisting, the vomiting, purging, and spasms ceased, a gentle perspiration appeared on the skin, and he recovered without another symptom of any kind. It was the most striking instance I ever saw of pseudo-cholera being cut short.—p. 207.

In the advanced stage he does not think it so useful, although if it do no good, it seems not to be injurious, and occasionally relieves the painful dyspnœa and oppression at the heart. It is, however, very difficult to get blood at this period; it flows from the arm in drops, and warm fomentations are often necessary even to procure these. According to Giacomini—

Blood-letting, as a rule, ought to be practised largely, with a view of preventing the phlebitis, the dilatation of the veins, their engorgement and their immobility. We say, as a rule—for if we wait until dilatation be effected and permanent, the blood only flows drop by drop, and all that is obtained only serves to empty the inferior part of the vein opened, without producing any advantage to the patient—we say, blood-letting ought to be large; for if the quantity extracted does not correspond to what is indicated the bleeding is of no effect, and, as the disease makes progress, inexperienced persons attribute to the bleeding the exasperation of the malady.—(*Annales, p.* 333.)

Bleeding, when it can be practised, therefore, seems not only theoretically valuable in order to remove the venous congestion, but when employed judiciously, has been found practically beneficial.

*Cold to the surface.*—Empirical practitioners, amongst whom we must place Dr. Milroy, naturally conceive, that when so much coldness of surface exists, heat is directly indicated. He says—

The first thing to be done is to have the patient at once stripped and enveloped in blankets. The application of bottles of hot water, bags of hot salt or bran to the feet, between the legs, and along the course of the spine, will always be useful in increasing the warmth of the general surface. This is a point of great importance; as the cutaneous circulation is all but arrested, and the blood is consequently accumulated in the internal viscera The sympathy between the skin and alimetary canal is kown to every one by experience. Cold feet will often cause severe pain in the stomach and bowels; and, on the other hand, indigestion and diarrhœa are almost invariably attended with a chilly state of the surface The removal of the exciting cause in either case will speedily relieve or altogether dissipate, the superinduced symptoms. How important then it must be, to act upon this therapeutic principle in a disease like cholera, in which the whole body is marbly cold, and the gastro-intestinal canal is so strangely and violently perturbed!—p. 43.

Yet this seems perfectly hypothetical, and constitutes an admirable commentary on the inutility of the empiric practice generally, which instead of seeking to remove the pathological cause of the disease, loses time in vainly endeavoring to alleviate the individual symptoms presented. How opposite are the statements of Parkes and Giacomini. For instance, Dr. Parkes says:—

Warm-baths, vapour-baths, and warmth applied in any way to the surface, never appeared to me to be of the slightest service in true cholera. The spasms were sometimes relieved, bnt the algide symptoms were almost invariably increased. The depressing effects of the warm-bath were somtimes marked and unmistakable. I have seen a man walk firmly to the bath, with a pulse of tolerable volume, and a cool but not cold surface, and in five or ten minutes have seen the same man carried from the bath, with a pulse almost imperceptible, and a cold and clammy skin. I cannot find in my notes a single case in which the warm-bath appeared beneficial. It is, indeed, unlikely that the attempt to restore warmth by these trifling means, when the grand source of animal heat is so disordered, can ever be successful. Several writers have also recorded their belief in the inutility of this measure.

Cold to the surface was a measure much more grateful to the patients than warmth. This might have been anticipated also from the way in whichthe bed-clothes are thrown off, so as to expose the surface freely to the air. The cold affusion, even in the last stage, two or thee hours before death, sometimes caused the pulse to become again perceptible. Perhaps the application of cold to the surface may affect the respiration in some way; the gasping inspiration which the shock of the falling water generally induces, may influence the circulation in the lungs, like the first impression of the cold air on the newly born infant. But unfortunately, after a short time, the reviving effects of the cold affusion disappear, and the case resumes it former course. The use of large fans and punkahs, causing a blast of air upon the body, seemed to me to be occasioally useful, and to be generally agreeable to the patient.—(*Parkes, p.* 209--211.)

Again Giacomini observes—

What seems wonderful and incredible is, that the cold bath during the algide period should be immediately followed by heat of skin, elevation of the pulse, cessation of the cramps, and fredom of respiration, as I have observed this very morning (7th July 1836.) In my opinion, therapeutics up to this time does not possess a more efficacious and prompt remedy wherewith to combat the cholera than the cold bath, the passage in the vessels being obstructed by vascular hyposthenics. It is important, however, that in this mode of treatment the cold application should not be alternated with warm, as the employment of these last may become very hurtful.—*Annales*, p. 333.

Cold baths repeated morning and evening have done prodigies; not only the heat and pulse have reappeared by this treatment; but bleeding, impractable until then, became possible, and the disease was overcome in the majority of cases most grave.—(*Ibid.* p. 334.)

We leave, then, our readers to judge whether an empirical or rational practice should be followed in the application of heat and cold.

*Injections into the Veins.*—We have already seen that medicines will not pass into the blood when taken by the mouth; their direct introduction therefore into the circulation by means of injection, although a bold practice, seems to be perfectly warrantable The immediate effects produced by saline injections of Dr. Latta of Leith, and Dr. Mackintosh of Edinburgh, under the idea that the salts in blood were defective, have been described by all who saw them to be most extraordinary, They dissolved ℥ss of muriate of soda, and ℈iv of sesquicarbonate of soda in ten pints of water, at a temperature varying from 106° to 120°Fh., which were injected slowly, half an hour being consumed in the process. After the injection of a few ounces, the pulse which had ceased to be felt at the wrist, became perceptible, and the heat of the body returned. By the time three or four pints had been injected the pulse became good; the cramps ceased; the body that could not be heated was rendered warm, and all other symptons were alleviated. These magical effects, however were not lasting. The dicharges continued, and the evacuations became even more profuse; the patient now relapsed into his former conditon, from which he could again be temporalily roused, by repetition of the injection; the amendment, however, was more transient, and death followed.

Dr. Parkes says, that in some cases which he witnessed in India, he did not see the temporary vivifying effects which generally followed the employment of these injections in Edinburgh; he therefore determined on trying some other agent.

I still thought that alkalies and salines seemed indicated by the evident occasional escape of the water and salts of the blood, and I fancied some benefit might result from an attempt to supply to the system a proteine compound; although of course, I could form no conception of the probable mode in which such an additon could be useful. I determined, therefore to inject into the veins an alkaline solution of albumen. I shall now detail, as briefly as the subject will permit, the few cases in which these injections were used. In all these cases I believed the patients doomed to an inevitable death. I did not consider myself authorized to try an experiment of so serious a nature on a man, while there yet remaind a chance of his rallying under the ordinary treatment. Consequently, I must premise, the plan was tried under the most unfavorable circumstances.—p. 219.

The solution injected was composed of sesqui-carbonate of soda ʒiv; chloride of sodium, ʒij; the albumen of one egg; and four pints of water, at a temperature of 98°Fh. The flakes of coagulated albumen were separated by filtration, and the fluid was slowly injected in five desperate cases. We regret that our space will not allow us to give the details of each. Suffice it to say, that there was, as in the case of purely saline injections, a marked temporary improvment, but they all ultimatly died. Dr. Parkes observes—

My own impression is still somewhat in favor of this practice. All these five cases were of the worst kind; there did not appear to be a chance of recovery for any of them under the ordinary treatment, and yet one was certainly carried through the cold stage, and, if differently treated during the consecutive fever, might have been completely cured. I think also that the alkaline solution might have been made weaker with advantage, and other ingredients might perhaps have been added.

The operation should I think, be again tried; it is very simple, it gives no pain, for in this stage of the cholera the skin is almost in sensible; and itcan do no harm to the patients, who are, in fact, doomed to almost certain death. The possible supervention of phlebitis in an after stage should, I think, be disregared; this disease is less formidable than cholera. The injection is not to cure cholera, but to restore and to sustain the circulation for some few hours, until the healing force of nature may repair the lesions of the blood and restore to the vitiated fluid its normal composition.---p. 237-9.

On the whole, we agree with Dr. Parkes in thinking, that injections into the veins, perhaps somewhat modified, deserve a more extensive trial.

